# Somatic genetic variation in healthy tissue and non-cancer diseases

**DOI:** 10.1038/s41431-022-01213-8

**Published:** 2022-10-27

**Authors:** Manuel Solís-Moruno, Laura Batlle-Masó, Núria Bonet, Juan I. Aróstegui, Ferran Casals

**Affiliations:** 1 Institut de Biologia Evolutiva (CSIC-UPF), Departament de Ciències Experimentals i de la Salut, Universitat Pompeu Fabra, Doctor Aiguader 88, Barcelona, Spain; 2 Genomics Core Facility, Departament de Ciències Experimentals i de la Salut, Universitat Pompeu Fabra, Parc de Recerca Biomèdica de Barcelona, 08003 Barcelona, Spain; 3 Department of Immunology, Hospital Clínic, Barcelona, Spain; 4 Institut d’Investigacions Biomèdiques August Pi i Sunyer (IDIBAPS), Barcelona, Spain; 5 Pediatric Infectious Diseases and Immunodeficiencies Unit, Hospital Universitari Vall d’Hebron (HUVH), Vall d’Hebron Institut de Recerca (VHIR), Universitat Autònoma de Barcelona, Barcelona, Spain; 6 Universitat de Barcelona, Barcelona, Spain; 7 Departament de Genètica, Microbiologia i Estadística, Facultat de Biologia, Universitat de Barcelona, Barcelona, Spain; 8 Institut de Biomedicina de la Universitat de Barcelona (IBUB), Universitat de Barcelona, Barcelona, Spain

**Keywords:** Medical genomics, Medical genetics

## Abstract

Somatic genetic variants have been studied for several years mostly concerning cancer, where they contribute to its origin and development. It is also clear that the somatic variants load is greater in aged individuals in comparison to younger ones, pointing to a cause/consequence of the senescence process. More recently, researchers have focused on the role of this type of variation in healthy tissue and its dynamics in cell lineages and different organs. In addition, somatic variants have been described to contribute to monogenic diseases, and the number of evidences of their role in complex disorders is also increasing. Thanks to recent advances in next-generation sequencing technologies, this type of genetic variation can be now more easily studied than in the past, although we still face some important limitations. Novel strategies for sampling, sequencing and filtering are being investigated to detect these variants, although validating them with an orthogonal approach will most likely still be needed. In this review, we aim to update our knowledge of somatic variation detection and its relation to healthy tissue and non-cancer diseases.

## Introduction

Mutation is the biological process that originates a genetic variant. Thus, somatic variants are caused by postzygotic mutations, those arising after the encounter of the spermatozoid and the ovule. They can occur from the beginning of the development of the organisms to any point during their life. Postzygotic mutations give rise to mosaicism, in which different cells of the same individual present dissimilarities in their genomes, in contrast to germline variants, inherited by the individuals from their parents and present in every cell of the body. To our knowledge, the first mention of the concept of the accumulation of somatic mutations, which were referred to as “ageing hits” on somatic cells, appeared in 1959 by Leo Szilard [1]. It represented the first remarkable effort that linked genetic damage and senescence. He developed his theory from a mathematical point of view and mentioned mutational agents such as ionizing radiation. Also, the concept of the somatic hypermutability in antibodies has been known since the 1980s. However, in the last 15 years, there has been an acceleration in the study of somatic variation, both in health and disease status, mostly because of the advances in next-generation sequencing (NGS) technologies.

Somatic mutations accumulate with time, with aged individuals presenting significantly more variants than younger ones. It is also well known that both extrinsic (tobacco smoke, alcohol, radiation) and intrinsic (cytosine deamination, oxidative damage, DNA replication errors) mutational agents contribute to the emergence of somatic mutations. Also, certain somatic variants are the causative agent of different diseases: many driver mutations have been described in cancer, and some of them cause monogenic diseases. For complex diseases, the link is harder to establish, although some studies have suggested that somatic variants may play an important role in neurological and other disorders. Recent technological advances allow a better characterization of somatic variation in human tissues. However, there are still many questions to be answered on its role and relative contribution to human disorders, for which it is first crucial to understand the mechanisms that govern somatic variation origin, distribution and transmission. In this review, while being virtually impossible covering the whole extensive and diverse literature on these topics, we aim to provide a description for understanding the current state of this research field, including the evolution of the different methodological strategies for the detection and analysis of somatic genetic variants, the knowledge of the presence and dynamics of somatic genetic variants in healthy tissue, and their effect on non-cancer disorders.

## Somatic variant detection

Heterozygous germline variants are present in half of the DNA molecules, which is reflected in peaks of similar height in Sanger sequencing or a presence in approximately 50 % of the reads for NGS. In fact, allelic imbalance, detected as a deviation from the expected 50 % variant allele frequency (VAF) across NGS reads, is a filter commonly applied during variant calling as a strategy to discard sequencing and mapping artefacts from true germline variants. In contrast, somatic genetic variants are usually found at VAFs below 50 % and the proportion of carrier cells in each tissue will condition the capacity of their detection. Sanger sequencing is unable to detect those somatic variants with VAF below ∼10 %, indistinguishable from the background signal. Also, variants with VAFs above ∼30 % can be misinterpreted as germline [2]. Alternatively, Sanger sequencing of multiple clones derived from individual DNA molecules can be used, although this approach requires a considerable amount of lab work. Therefore the use of these strategies is limited to validate or to better approach the frequency of candidate mutations, the accuracy of which will depend on the number of sequenced clones, rather than for new variant discovery.

**Fig. 1:**
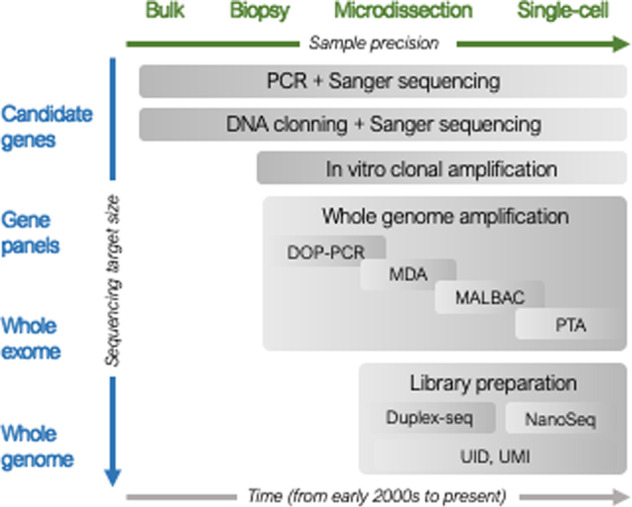
Sample types (green), sequencing approach (blue) and methodology (grey) used to study somatic variants along time. Gradient in each box approximately represents the intensivity of use at different periods. DOP-PCR degenerate oligonucleotide-primer PCR, MDA multiple displacement amplification, MALBAC multiple annealing and looping-based amplification, PTA primary templatedirected amplification, UID unique identifiers, UMI unique molecular identifiers.

NGS overcomes most of these limitations, especially when high sequencing depth is achieved. For this reason it has been the main strategy employed during the last years to discover and characterize somatic variants. Different strategies have been developed, which mainly differ according to sample obtention and type as well as the sequencing approach (Fig. 1). On the sample nature, sequencing from bulk tissue was initially the standard approach with the constraint of consisting in a mixture of cell populations with multiple clonal origins. In this sense, microbiopsy and microdissection resulted in higher purity of the sample although it will still show cell-type heterogeneity. Alternatively, sequencing at the single-cell level presents the theoretical possibility of identifying any single mutation in a cell, representing one step forward towards the understanding and characterization of the somatic genetic variant load in healthy tissues. Several technologies are available for single-cell isolation: micromanipulation, serial dilution, Fluorescence Activated Cell Sorting (FACS), Magnetic-Activated Cell Sorting (MACS), Laser Capture Microdissection (LCM), or microfluidic devices. FACS and MACS allow the individualization of dissociated single cells that have been previously marked, the first with fluorescence and the second with antibodies conjugated to magnetic beads, which confer to these methodologies a higher specificity. In contrast, LCM is a low-throughput method that can be used with fixed and live tissue, although the capture can include more than one cell and it requires advanced technical skills. Finally, microfluidic or droplet devices are gaining territory as emerging single-cell isolation methods [3].

Once cells have been isolated, one of the issues that single-cell genomics methods encounter is the relatively large amounts of genetic material required in current sequencing methods, in comparison to the low amount of DNA present in a cell. Two main strategies have been implemented to face this limitation: single-cell isolation followed by whole-genome amplification (WGA), or clonal amplification of single cells in culture for DNA extraction plus sequencing (Fig. 1). The goal of WGA is to amplify the genome of a single cell with the highest precision while minimizing the amplification bias. Initially, this methodology was based either on the ligation of adaptors that were used as specific primer sites for PCR amplification, or on the use of primers targeting repeated sequences in the genome. Improved techniques were designed including degenerate oligonucleotide-primer PCR (DOP-PCR), multiple displacement amplification (MDA), and multiple annealing and looping-based amplification cycles (MALBAC) [3]. DOP-PCR is an exponential PCR method that uses random primers and a thermostable polymerase which has a higher error rate compared to the thermolabile enzymes. As said, one of the improvements of MDA is the use of isothermal polymerases that have a reduced error rate at the expense of having less uniformity. In between both approaches, MALBAC starts with an isothermal preamplification followed by PCR amplification. An important caveat of these techniques is the mutation rate, up to 1–40 mutations per 100 kb [4], which needs to be considered in the variant calling process and subsequent analyses. Primary template-directed amplification (PTA) is an improved alternative that takes advantage of the low error rate of the polymerase used in MDA with some modifications in the amplification [5]. The process is quasilinear as the amplification occurs mostly from the primary template. The result is that errors have less propagation to subsequent amplicons. Furthermore, their developers claim a recovery of 95 % of the single-cell genomes in a more uniform representation.

As an alternative to WGA, in vitro clonal expansion of single cells relays in the intrinsic replication mechanisms of the cell division. Classical 2D culture was one of the earliest methods used for this purpose yet it is difficult to achieve for some cell types, particularly from a single cell. Induced pluripotent stem cell (iPSC) culture is another option. However, the culture conditions and environmental agents can be a selective bottleneck to a group of cells and can induce de novo mutations during culture [4]. In contrast, organoid culture technologies have the advantage of being compatible with multiple human cell types, and long-term genome-stable cultures can be obtained. This approach is more time and resources consuming, but still more accurate and sensitive. In addition, it produces viable cells that can be used for further experiments.

As for the library preparation protocols, methods based on the use of unique molecular identifiers (UMI) also contribute to reducing error rates. Duplex sequencing was one of the first methods to include tags to independently barcode each strand of a DNA duplex so that true mutations have to be found at the same position in both strands, and presenting a theoretical background error rate claimed of less than 10^−9^ errors per base pair [6]. Another alternative is the Safe-Sequencing System (Safe-SeqS) which incorporated the use of unique identifiers (UID, an equivalent to UMI) [7]. These strategies were the basis of many new NGS methods such as BotSeqS or its most recent derivative NanoSeq [8] which presents error rates lower than five per billion base pairs. Both methods take advantage of the use of Duplex-Seq Adaptors (IDT) that mark individual molecules and strands. NanoSeq also reduces the substitution imbalances that are still present in standard BotSeqS protocols estimated to be caused by the end repair and nick extension processes in the library preparation. As an alternative, NanoSeq replaced the sonication fragmentation and end repair by restriction enzyme fragmentation or sonication plus exonuclease blunting and modified the A-tailing step to avoid errors from nick extension.

Regarding the sequencing approach, studies to detect somatic variants have used deep sequencing of target genes, whole-exome sequencing (WES) and whole-genome sequencing (WGS) (Fig. 1). Because of the need of achieving high sequencing depths, targeted sequencing and WES approaches have been extensively used in the analysis of tissue samples and biopsies [9, 10] as in the case of cancer analysis from tumours. These studies are restricted to candidate genes, which in addition to focusing on the subsequent analysis and interpretation, have the advantage of providing high sequencing depths with more affordable costs. Other works have used genomic approaches (WES, WGS and/or RNASeq) to the study of somatic genetic variation in healthy tissues [11–14]. In most of these cases, samples are generated by microdissections or single-cell analysis, which partially compensates for the smaller sequencing depths achieved in WGS compared to targeted analysis. Of importance, DNA sequencing after clonal expansion in cell culture or developing organoids [4] can be used as an approach to uncover all genomic regions and avoid the possible biases of whole-genome amplification from a single cell, where each allele is represented only once. Finally, WGS also confers the opportunity to expand the analysis more accurately to copy number variation (CNV) in addition to SNVs and indels. In that line, comparative genomic hybridization has also been used for structural variants detection from single cells [15].

Once sequencing data is generated, several specific bioinformatics algorithms have been created for somatic variant calling, such as CaVEMan, EBCall, LoFreq, SomVarIUS, Strelka2, VarDict and VarScan2. However, the comparison of the results generated by these tools has revealed, in general, poor levels of overlap [16] given the different algorithms and original purposes of such callers. To resolve this issue, pipelines implementing different combinations of the filtering strategies have been proposed. After somatic variant calling, and depending on the generated data, the implementation of a set of filters is mandatory to exclude false positives. Because of the modification of the frequency and imbalance thresholds filters, the number of candidate genetic variants is usually very high and true somatic variants need to be discriminated from germline variants and sequencing, mapping or calling errors. The commonly used filters are those based on mapping and calling quality, expected VAF, comparison to variants called in other samples, allelic imbalance, position at the beginning/end of the reads, etc [17]. Finally, somatic genetic variants should be validated by using a different sequencing approach. For this purpose, amplicon-based deep sequencing is being extensively used in current studies, also providing a more accurate estimation of the VAF in the sample. Of note, the simultaneous analysis of different amplicons encompassing the same genetic variant is recommended to avoid inaccurate frequency estimations due to PCR biases [2].

## Somatic variants in human development and adult tissues

Somatic variants have been traditionally studied in the context of cancer. More recently, enhanced by the development of NGS technologies, the presence of somatic genetic variants in healthy tissues is being characterized. Many of the studies use different combinations of the main approaches for sample obtention and somatic variant characterization (Fig. 1) and are performed on healthy tissues obtained in the context of cancer analysis. While providing valuable information on particular tissues and organs, this methodological diversity makes the studies hardly comparable among them in terms of somatic variant distribution, frequency, or differential mutation rates across tissues. Recently, some studies performed extensive multi-tissue analysis allowing to perform a direct comparison, which confirmed a non-uniform distribution of somatic variation across somatic tissues [8, 14]. The results showed a few tens of single base substitutions (SBS) per year with up to two- to fivefold differences among tissues, which might be independent of the cell division rate [8]. The mutation rates obtained by several studies exploring specific cell types and tissues are in concordance with this observation. These rates are translated in a load from hundreds to a few thousands of somatic SNVs in a whole genome (e.g. 200–2000 in oesophageal epithelium [10] or around 1500 in neurons [18], and up to 1500–15,000 in colonic crypts [12] which present the higher substitution rates across the tissues analyzed). In contrast, spermatogonia showed a lower rate, similar to the 1.35 SBS per year inferred from trio analyses [8].

Germline cells represent a particular tissue regarding the analysis of genetic mosaicism. Contrary to somatic variants found in non-germline tissues, genetic variants in gonadal or gonosomic mosaicisms can be transmitted to the next generations. These de novo variants are known to contribute importantly to the load of genetic disorders in human populations. In that regard, there exists an important level of asymmetry in female and male contributions with 80 % of de novo SNVs being inherited from the father, probably because of the elevated proliferation of spermatogonia compared to oocytes with no mitotic activity. This explains the increase of the genetic variants transmitted by males to the offspring with age, because of replication and mitotic errors in sperm, while other changes such as aneuploidies are more present in oocytes [19]. Sperm mosaicism has been classified into different types according to their origin in primordial, spermatogonia or sperm cells. While the number of mutations occurring in the earlier developmental steps and later at the sperm cell levels are constant across time, mutations in spermatogonia stem cells increase with paternal age. This also has important consequences on the risk of recurrent transmission to offspring which is negligible for more recent mutations affecting the latest steps of sperm maturation but higher if occurred earlier, especially at the level of primordial germ cells [19].

The somatic mutation rate also does not remain constant over time, with higher rates in the first developmental stages and foetal life compared to post-natal ones. Several studies have used somatic variants as barcodes to define cellular phylogenies and study cellular dynamics in the embryo and development, revealing an increase of the mutations per cell division in the first two or three cell divisions. The mutation rate is also increased in several foetal tissues analyzed, up to fivefold higher than the same tissues in adulthood. Many factors can contribute to this excess of somatic mutations during early developmental stages including a reduced time for DNA repair because of the shorter duration of cell cycles or relaxation and dilution of the reparatory mechanisms maternally inherited in absence of transcriptional activity in the first egg divisions [20]. The mutational signatures SBS1, a cell division clock signature and associated with the deamination of 5-methyl cytosine that happens before DNA replication [21], and SBS5, associated with the ageing process, are ubiquitously observed although with distinct relative contributions across tissues. SBS18, caused by oxidative damage, is also quite common across many tissues. Contrary, other signatures are restricted to particular cell types pointing to a different relative contribution of mutational mechanisms and exposures. For instance, SBS7 is associated with UV light exposure and found in the skin [9] and individual melanocytes [22]; SBS4 is associated with tobacco smoke and found in smokers’ and ex-smokers’ lungs [11]; and SBS16 is associated with alcohol consumption in the oesophagus.

In addition to studies analyzing somatic variation across different tissues, many works have focused on particular cell types. Along with these studies human tissues are asymmetrically represented, with a higher representation of tissues more easily accessible or obtained in the context of routine biopsies or surgeries, as well as because of the major interests that some tissues have generated in the research community (e.g. brain). The epithelium is among the most represented tissues, although with a considerable variety of types analyzed including eyelid epidermis, oesophageal and bronchial epithelium, liver or colonic crypts, endometrial glands or urothelium. Some of these tissues are particularly exposed to mutagenic agents such as sunlight, tobacco and alcohol, with a confirmed role in the increase of somatic mutations. Somatic variants in sun-exposed tissues present a pattern similar to that typically related to UV exposure, although it is higher in intermittently sun-exposed skin (back and limbs) than in sun-exposed skin (face) probably related to different mutation rates, DNA repair mechanisms or turnover frequency [22]. The effect of tobacco on increasing the mutational load has been shown in oesophageal and bronchial epitheliums. Positively selected or mutationally enriched genes have been found in some of these tissues, such as *NOTCH1* (and other Notch family members) in eyelid or oesophagus epithelium, or *PIK3CA* in colorectal crypts and endometrial glands. Skin fibroblasts have also been analyzed, showing a rate of UV-induced somatic variants which do not correlate with age [23]. Also, skin tone was significantly impacting the load of somatic variants, with European ancestry individuals showing a higher mutational load in skin fibroblasts and melanocytes, probably due to the protective effect of melanin [24].

Brain and nervous system cells have also been analyzed by several studies, mostly in the context of analysis of neurodegenerative disorders. Neurons are terminally differentiated post-mitotic cells, thus, somatic variants in more than one cell will have occurred during development. In contrast, mutations occurred later will affect only the original cell. The observed enrichment in coding exons with a strand bias supports this, as the pattern is expected for mutations arising in the transcription process [18]. In that line, the study of two brain areas, the prefrontal cortex and the dentate gyrus of the hippocampus revealed interesting features such as differences between the regions, or a mutation pattern changing with age. For example, among younger individuals, C > T mutations (Signature B) are the most common, but their fraction decreased with time. Several studies have also attempted to detect somatic structural variants in the human brain, as the effort to create a brain CNV atlas for neurotypical individuals revealing important levels of variation across individuals and an inverse correlation with age [25] contrary to the hypothesis of the accumulation of SV due to ageing [26] which detected variants with higher VAFs (up to fivefold) in older people. Finally, a yet underexplored branch of somatic structural variation is the role of transposable elements. Using single-cell studies, although Evrony et al. initially showed that LINE1 insertions were not a source of mosaicism in neurons, the same authors later reported clonal LINE1 retrotransposition events in the human brain [27]. Upton et al 2015 [28] described similar results in hippocampal neurons. This shows the rapid evolution of the field and encourages the debate about the comparison of somatic events using different tissues and technologies.

## Somatic variants in aging

We accumulate somatic mutations throughout life, a fact supported by a great number of studies, as these in the epithelium of oesophagus [10, 29]; endometrium [14] lung [11]; colon [12] and bladder [13]. Also in bulk skin [9], individual melanocytes [22]; liver [30]; neurons [31] and blood [32, 33]. This happens in fully differentiated cells, but it has also been observed in adult stem cells of the small intestine, colon and liver [34]. In fact, even the speed of accumulation of these mutations is accelerated when the aging process is boosted in mice [35]. Whether the accumulation of somatic variants is a cause or consequence of the aging process is still under discussion [36].

An important process linked to ageing is muscle decline, especially in skeletal muscle which facilitates the mobility of bodies. In that sense, somatic variants were studied in the satellite cells of muscles, where they have been observed to be accumulated with age, also in exons and promoters. The authors suggest that this accumulation is a driving force behind the decline of this type of muscle [37]. We are not aware of any work studying somatic variation in smooth muscle. Along with the physical decline of the ageing process comes the cognitive one. We have already mentioned a few works exploring somatic mutations in neurons [31], in which the accumulation of these variants with age has been described and linked to ageing and neurological diseases. Actually, neurodegenerative diseases are mostly diagnosed in elderly individuals, a topic developed later in this review.

## Somatic variants in clonal haematopoiesis

Haematopoiesis is, briefly, the process by which mature blood cells are formed in the bone marrow from haematopoietic stem cells (HSCs). By studying the whole genomes of HSCs and downstream multipotent progenitor cells (MPPs) clones, from people of different ages, an accumulation of somatic SNVs in a linear fashion has been observed [38] at a ratio of 14.2 new base substitutions per year. Of note, indels did not seem to correlate with age. Somatic variants have been widely studied in blood due to the easy sampling, especially with the focus on clonal haematopoiesis (CH). We understand CH as the phenomenon by which a single clone dominates the haematopoiesis process and it is overrepresented in blood. CH is related to ageing and several malignant pathologies. Among the several genes reported to be linked to CH [33, 39] we highlight *DNMT3A*, *ASXL1* and *TET2*, three epigenetic regulators, as genes recurrently found to harbour somatic variants in individuals with CH. Recently, and taking advantage of a dataset generated for cancer research with around 12,000 blood-cancer paired samples, the list of genes under positive selection in CH has been expanded up to almost 70 [40].

In the past, CH has been described as more common than previously observed in people older than 65 [32]. In that study, genetic drift was proposed as the process behind CH, something that also occurred with clonality in other tissues such as the stomach [41] and intestine [42]. However, in a metastudy published in 2020, in which several of the aforementioned big datasets were reanalyzed, positive selection was seen as the main driving force of CH, the authors argue, among other reasons, that VAFs observed were too high to be caused by drift [43]. The relationship between CH and disease is explored in the previous works. Not surprisingly, different haematological cancers are the most linked diseases to this process. Within CH we find a linked term: clonal haematopoiesis of indeterminate potential (CHIP). It is defined by the existence of cancer-associated clonal mutations, with VAF > 0.02, in blood cells or bone marrow, but without having an actual malignancy [44]. The probability to develop cancer in individuals with CHIP is low, and it is calculated to be increased by this condition by around 0.5 %-1 % per year. Also, the presence of CHIP almost doubles the risk of having coronary heart disease and makes the risk of myocardial infarction four times higher [45].

## Somatic variants in monogenic diseases

In the same way as germline variants, somatic variants can be the cause of monogenic diseases if they reach a sufficient VAF in the affected organ or tissue. This is especially relevant for diseases with an autosomal dominant inheritance pattern, or those X-linked in men, where only one altered copy of the causal allele is needed to develop the disease. The first study proving the direct impact of somatic variants in disease is more than 25 years old [46] which described mutations in the *PIGA* gene as causative of paroxysmal nocturnal haemoglobinuria.

A very remarkable example of somatic variants in monogenic disorders are inborn errors of immunity (IEI), with growing evidence of the role of genetic mosaicism in recent years [47]. In addition to particular mechanisms such as clonal hematopoiesis that can increase the frequency of a certain genetic variant, a methodological bias because of the accessibility and universal use of the affected tissue, blood, could also contribute to an overrepresentation of IEI among the reported cases. The pioneer studies reporting the first somatic variants associated with IEI are from 2004 [48] and 2005 [49], in which the *NLRP3* (former *CIAS1*) and *FAS* genes were identified as harbouring causative somatic variants. Interestingly, *NLRP3* is nowadays the gene with the greatest number of reported somatic variants linked to autoinflammatory syndromes [50], a family of disorders classified within IEI. On the other hand, the *FAS* gene, underlying autoimmune lymphoproliferative syndrome (ALPS), is an interesting example of various genetic mechanisms converging in similar phenotypes. *FAS* causes ALPS in patients who harbour pathogenic germline variants, somatic variants, and a combination of them [51]. Up to 20 % of the cases are caused exclusively by somatic variation, which is restricted to a specific cell population and can be detected by NGS [51]. Other outstanding cases are somatic variants in *NOD2* causing Blau syndrome [52]; *NLRC4* causing NOMID (neonatal-onset multisystem inflammatory disease) [53]; and *UBA1* associated with the late-onset VEXAS (vacuole, E1 enzyme, X-linked, autoinflammatory, somatic) syndrome, a severe X-linked disorder affecting males and restricted to cells of the myeloid line [54]. We could also extend the list of genes harbouring somatic variants being causative of an IEI to *BTK*, *ELANE*, *IL2RG*, *NRAS*, *PIK3CD*, *STAT3* and *WAS* [2]. Interestingly, we have recently reported that those causal genetic variants are detectable from moderately high sequencing coverages in WES data from blood, by adapting the variant calling bioinformatics pipelines and establishing a set of stringent filters [55]. Thus, it could be recommendable considering the detection of somatic variants in non-diagnosed cases after NGS, which if restricted to a set of candidate genes can be a feasible and affordable approach.

The list of reported cases of diseases includes other organs and tissues beyond blood, such as Alport syndrome, in which somatic variants in *COL4A3* can impair the function of kidneys [56], or Huntington’s disease with a somatic expansion of the CAG triplet in the *HTT* gene in the brain [57]. We also want to highlight the existence of several diseases in which only somatic variants, and not germline, have been described. In these cases, the most feasible hypothesis is that germline variants would be highly lethal. This is supported by the severe effects of the somatic alterations, such as somatic-derived hemimegalencephaly [58].

Of interest, a somatic mutation could also reverse a pathogenic variant acting as a spontaneous reversion of germline inherited disease [59]. These new variants may confer an advantage to the cell and would be targeted by natural selection and increase in frequency. Therefore, the presence of the new variant may have a positive impact on the phenotype of the affected individuals, who can experience significant clinical improvements. This is called somatic genetic rescue and has been described in various diseases, among which many IEI [60]. The presence of such events has important diagnostic implications, as it could explain unexpected clinical improvements, and also therapeutic implications, as an in vivo proof of the potential effect of gene therapy.

## Somatic variants in complex diseases

The study of the genetic aetiology of complex disorders was traditionally focused on the role of common genetic variants identified by linkage and association studies. Later, NGS allowed exploring the role of rare genetic variation thanks to the development of new statistical approaches mostly optimized to identify the enrichment of this type of variants in patients compared to controls, or even to explore the role of de novo genetic variants in particular individuals that would therefore better fit to a Mendelian inheritance. The de novo scenario has been particularly explored for neurodevelopmental disorders, where these mutations would be unlikely to be observed as germinal because of the low reproductive rates of affected individuals [61]. Similarly, new genetic variants arising during development or even in adult neurons could also contribute to the development of these disorders.

Several works propose a possible relation of somatic variants to neurological disorders such as Alzheimer’s disease [62] or Parkinson’s disease [63]. Also, individuals with a neurodegenerative condition showed an increase in the number of somatic SNVs in comparison with matched healthy individuals of similar age.

A somatic copy number alterations was found to be responsible for the neuropsychiatric disease [64] at VAFs below 20 %. Also, in autism spectrum disorder, structural variants affecting 3–70 % of the cells were detected by microarrays [65]. Interestingly, in this case, the event size positively correlated with the severity of the patients’ symptoms [65]. Recently, following the idea that LINE1 does play a role in the diversity of the neuron’s genome, Zhu et al. corroborated the possible pathogenic effect of retrotransposon insertion on neurologic disease [66].

As mentioned before, CH is related to a group of complex diseases, also beyond cancer. For instance, there is a relationship between chronic ischaemic heart failure and the mutations causing the CH [67]. The somatic variant landscape has been also studied in endometriosis [68], in which the authors performed WES on 13 endometriotic and 11 normal endometrial epithelium samples. They also performed targeted sequencing on a larger validation dataset of 94 endometriotic epitheliums from 45 individuals and 71 normal epitheliums from 29 individuals. They discovered two oncogenes, *KRAS* and *PIK3CA*, to be the most frequently mutated in both scenarios, but with VAFs significantly higher in endometriosis. Thus, the authors suggested a potential role in the development of endometriosis of the generated clonal expansion due to the presence of these cancer-associated mutations.

Following a previous publication on colonic crypts [12], and using the generated samples in that study as healthy controls, inflammatory bowel disease (IBD) has been explored. In this new study, WGS was performed on 446 crypts from 46 IBD patients with a median coverage of 18.2× [69]. They sequenced 28 ulcerative colitis patients and 18 Crohn’s disease patients and described an average of ~2.4-fold increase in the mutation rates of IBD patients compared to controls, and reported no significant differences between the patients of both diseases. They observed that around 80 % of the increase in mutation burden is explained by mutational signatures also present in the healthy colon, pointing to an acceleration of the normal age-related mutational processes. Besides this, the clonal expansion found in patients with IBD is much larger than in healthy individuals, with an important number of clones bigger than 2 mm. They also found signatures of positive selection in genes *ARID1A*, *FBXW7*, *PIGR* and *ZC3H12A*, as well as in some of the IL-17 and Toll-like receptor pathways, suggesting differences in selection mechanisms in IBD colons. Finally, the authors suggest a potential causal role of somatic mutations in IBD. The case of ulcerative colitis has also been studied in another work, in which WES was performed on 76 clonal human colon organoids [70]. Similarly, the authors observed an accumulation of somatic mutations in genes of the IL-17 signalling pathway such as *NFKBIZ*, *ZC3H12A* and *PIGR*. These genes are not particularly related to colorectal cancer.

## Conclusions

The number of evidences of the role of postzygotic mutations in non-cancer diseases is growing in parallel with the development of experimental and analytical methodologies to detect and characterize somatic genetic variants. Sampling methods are being refined allowing the characterization of genetic variants at the single-cell level in an increasing number of cell types. Simultaneously, bioinformatics pipelines are being adapted to different experimental designs to identify real somatic variants while discarding false positives generated by sequencing, mapping and mostly calling artefacts because of the relaxation of allelic imbalance thresholds. Of importance, a proper biological interpretation of the results can only be done based on the understanding of the presence and distribution of somatic genetic variants in healthy tissue, which provides a baseline for the analysis of somatic variation in disease. While there exist some commonalities across tissues as the accumulation of somatic variants with age and exposure to external agents such as tobacco, alcohol or sun radiation, we should ideally consider the specificities and different mutagenic patterns shown by each cell type.
